# Novel Procedures for Evaluating Autism Online in a Culturally Diverse Population of Children: Protocol for a Mixed Methods Pathway Development Study

**DOI:** 10.2196/55741

**Published:** 2025-02-11

**Authors:** Venus Mirzaei, Jeanne Wolstencroft, Georgia Lockwood Estrin, Eleanor Buckley, Shermina Sayani, Panos Katakis, Reena Anand, Tessa Squire, Eleanor Short, Paige Frankson, David Skuse, Michelle Heys

**Affiliations:** 1 Specialist Children & Young People's Services East London NHS Foundation Trust London United Kingdom; 2 Great Ormond Street Institute of Child Health University College London London United Kingdom; 3 School of Psychology University of East London London United Kingdom; 4 School of Health and Wellbeing College of Medical, Veterinary and Life Sciences University of Glasgow Glasgow United Kingdom; 5 Raigmore Hospital NHS Highland Inverness United Kingdom; 6 School of Life and Medical Sciences University of Hertfordshire Hatfield United Kingdom

**Keywords:** autism, child, telehealth, co-development, feasibility, acceptability, assessment, diagnosis, online, evaluation, diagnostic, intervention, pilot implementation evaluation study

## Abstract

**Background:**

Current autism assessment procedures are costly and resource-intensive. The COVID-19 pandemic accelerated the adoption of telemedicine, highlighting the benefits of innovative diagnostic tools. Telemedicine-based pathways could enhance accessibility and equity in autism diagnostics.

**Objective:**

The Children with Autism Technology Enabled Assessment (CHATA) project aims to develop and pilot an open-source autism diagnostic pathway for children up to 5 years old, delivered through telemedicine. The pathway is designed to be culturally and linguistically adaptable, increasing its applicability to diverse populations and integrating with existing National Health Service digital systems.

**Methods:**

Initial pathway development was informed by systematic evidence reviews, coproduction, and mixed methods usability. CHATA comprises 2 key elements: online self-completed standardized autism questionnaires and a structured online interview and observation by a trained clinician. Out of 60 families near the top of the local waiting list will be invited to participate in the pilot evaluation, assessed using both the CHATA and usual assessment pathways. Sensitivity and specificity will be calculated by comparing the diagnosis of autism through CHATA with usual care. Quantitative usability assessment will be gathered from all families using the System Usability Scale (where a mean above 68 indicates above-average usability). A subset of CHATA assessments will be reviewed for interrater reliability (measured by the Cohen κ for categorical data [diagnosis present or absent], with values indicating the level of agreement; eg, <0 indicating no agreement, 0.61-0.80 indicating substantial agreement). Qualitative data on acceptability, feasibility, and usability will be gathered from semistructured interviews with a subset of families and health care providers. We will recruit 60 families for the main pilot study (including the usability testing) and 10-15 participants for the qualitative substudy. Data will estimate CHATA’s diagnostic accuracy, validity, reliability, usability, and acceptability. Patient and public involvement will be integral throughout. The study will take place in a socio-economically deprived, ethnically diverse inner-London Borough within a community-based child health National health service responsible for the Autism assessment of children and young people up to the age of 13 years.

**Results:**

Ethics approval was received in June 2023 (Research Ethics Committee reference 22/LO/0751; IRAS project ID 320499). Data collection commenced in April 2023 and completed in October 2024. Project end date is March 2025. As of November 2024, we had enrolled 57 participants to the pilot study and 12 to the qualitative substudy.

**Conclusions:**

The CHATA project aims to establish a novel, culturally sensitive, equitable, and accurate online autism assessment pathway. By addressing geographical and linguistic barriers, this pathway seeks to reduce service costs, shorten waiting times, and promote equity in autism diagnosis. The procedures developed are expected to be generalized to other populations nationwide.

**International Registered Report Identifier (IRRID):**

DERR1-10.2196/55741

## Introduction

Autism spectrum disorder (ASD) is a complex neurodevelopmental condition marked by difficulties in social communication, restricted and repetitive behaviors, and sensory sensitivities [1]. Despite a significant increase in autism prevalence in the United Kingdom among children aged 10-14 years, only about 1 in 190 children are diagnosed during the preschool years [2]. Consequently, many children are not identified or assessed until they have been in school for several years, leading to educational disadvantages. As of December 2023, over 170,000 people in England were awaiting an autism assessment, with 85% waiting beyond the recommended 13 weeks [3]. While data on waiting lists for older children in child and adolescent mental health services are available, equivalent data for preschool assessments is less reliable [4]. However, anecdotal evidence suggests lengthy waiting times of 36-42 months for preschool appointments, with some regions having no availability at all. Autism can be reliably identified by 24 months [5], but the average diagnosis age is >5 years in Europe and North America [6,7].

Structural inequity exists in autism assessment. Girls, and people from ethnic minorities or lower socioeconomic groups, receive support later than their peers, if at all. The 2024 Child of the North (UK) report found that ethnicity and associated language barriers play an important role in determining who gets assessed; overall, children of white heritage are substantially more likely to receive an autism diagnosis than children of Asian heritage [8,9].

Traditional autism evaluations involve developmental history, current behavior descriptions, and direct observation of social interaction skills. The COVID-19 pandemic has increased interest in using telehealth for preschool autism assessments. Various tools and methods are used in telehealth, including video-based observations, digital questionnaires, and online behavioral checklists. These tools can be used for both live (synchronous) and recorded (asynchronous) assessments, providing flexibility in how evaluations are conducted. Telehealth offers benefits like flexible scheduling, increased accessibility, fewer no-shows, optional audio and video recording, reduced travel and costs, fewer room bookings, and lower environmental impact [10]. Families in rural or underserved areas can now access diagnostic services more easily through remote assessments [11]. In addition, telehealth can significantly reduce wait times by enabling quicker initial screenings and follow-ups, which is especially beneficial given the high demand for autism assessments and the limited number of specialists available [11]. Our recent systematic review found that Telehealth assessments are as accurate as in-person assessments, with over 80% diagnostic agreement [12]. Online asynchronous parental reports and behavioral observation tools also show high validity and reliability, with agreement rates of 82%-88% compared with in-person assessments.

However, telehealth also faces several limitations. Technological barriers are a significant issue, as not all families have access to the necessary technology or reliable internet connections [13]. This can create disparities in who can benefit from telehealth services. In addition, there is a need for more training for clinicians to effectively use telehealth tools and ensure consistent and accurate assessments [14]. The standardization of telehealth procedures is still evolving, which can affect the reliability of these assessments. Equity issues are another concern. Telehealth may not be equally effective for all populations, as cultural and language differences can impact the accuracy of assessments. Moreover, telehealth tools may not be validated for diverse populations, potentially leading to biased outcomes.

We have developed a telemedicine assessment pathway - Children with Autism Technology Enabled Assessment (CHATA) that combines both asynchronous (parent online questionnaires) and synchronous (online clinical assessment) elements. Designed currently for children up to 5 years old, it has been developed to optimize linguistic and cultural accessibility. Given the potential limitations of the telemedicine approach, refining approaches such as CHATA and conducting pilot trials are crucial. These efforts can help improve the efficiency of autism screening and diagnosis by developing more reliable and user-friendly telehealth tools. They can also address disparities in telehealth access and effectiveness, ensuring that all children, regardless of their background, receive accurate, and timely diagnoses. Furthermore, refining CHATA can contribute to the standardization of telehealth procedures, making them more consistent and reliable across different settings. Ultimately, this study aims to enhance the efficiency and equity of autism assessments for children. Our objectives are to refine the pilot version of the CHATA diagnostic assessment for preschool children with suspected autism, evaluate its usability, acceptability, feasibility, and interrater reliability, and estimate its sensitivity and specificity.

## Methods

### Study Design

**Figure 1:**
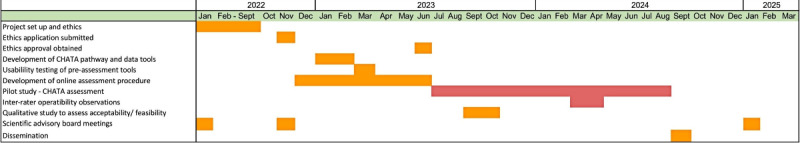
Study timeline. CHATA: Children with Autism Technology Enabled Assessment.

This mixed methods study integrates pathway refinement, pilot evaluation, and implementation, using both qualitative and quantitative approaches. It focuses on usability, acceptability, feasibility, clinical validity, and reliability. The research framework is grounded in Behavioral Science and Participatory Action Research [15]. This approach captures diverse challenges and resilience factors among families, addressing the limitations of solely using survey questionnaires. Qualitative data will enrich quantitative findings, providing a comprehensive understanding of individual experiences. The study timeline is provided in Figure 1.

### Study Setting

The study will take place in the London Borough of Newham (LBN), UK, known for its high ethnic and linguistic diversity, with approximately 78% of the population from ethnic minority groups and over 140 spoken languages. LBN is also among the most deprived areas in England, with over 50% child poverty. The East London National Health Service (NHS) Foundation Trust provides community child health services, including the Children with Autism in Newham–Diagnosis Service (CHAND) for children up to 13 years old. Due to underfunding and increased demand during the COVID-19 pandemic, over 1200 children are currently awaiting assessment by CHAND (personal communication as of June 14, 2024).

### Patient and Public Involvement

To inform the initial development of CHATA and this pilot study protocol, 2 patient and public involvement (PPI) workshops were held with 8 parents of children who had online autism assessments during the COVID-19 pandemic. The group included 5 Asian or Asian British parents and 3 Black or Black British parents, 1 requiring an interpreter. While most preferred face-to-face appointments, they accepted online assessments for reduced waiting times. A “Parent Voice” representative, an Asian parent of children with autism is a named co-investigator, who will participate in regular meetings, assist with study design, and analysis, and provide ongoing feedback.

### Stakeholder Engagement

A Scientific Advisory Board will guide the development of the online autism assessment pathway, meeting biannually with regular email updates. Additional engagement with the National Institute for Health and Care Excellence guideline stakeholders will explore novel provisions. Study outcomes will be disseminated through an event with local clinical service stakeholders, and key findings will be shared through the NHS Trust website. In addition to scientific manuscripts and presentations, a pamphlet will publicize the study’s outcomes and policy implications.

### Usual Autism Pathway Procedures in Study Setting

The usual care pathway in LBN, referred to as the “CHAND reference pathway,” currently includes the following steps:

1. Referral: made to CHAND.
2. Triage: conducted by a multidisciplinary team (MDT) based on referral information, resulting in 1 of 3 outcomes: acceptance, request for additional information (eg, school report), or rejection.
3. Assessment decision: if accepted, the triage team determines whether the case is straightforward or complex based on demographics (eg, age) and clinical factors.
4. Assessment: straightforward cases are booked to see a pediatrician or senior speech and language therapist alone. Complex cases are booked for a multidisciplinary assessment involving at least 2 clinicians (eg, pediatricians, speech, and language therapists, psychologists, and occupational therapists). One clinician conducts an Autism Diagnostic Observation Schedule, Second Edition (ADOS-2) assessment [16], and the other takes a case history. If a diagnostic outcome is not reached, the case is discussed at a wider MDT meeting, including representatives from pediatrics, speech and language therapy, occupational therapy, and psychology. Additional assessments may include a school visit, a Diagnostic Interview for Social and Communication Disorders with parents [17], or an informal observational assessment to evaluate mental health difficulties or masking in girls.
5. Feedback and follow-up: diagnostic feedback and psychoeducation about autism and autism-specific supports are provided. Local signposting and invitations to postdiagnosis group workshops or one-to-one sessions with a parent connector are offered.

The service is diagnostic only and does not provide postdiagnosis support or interventions. Onward referrals for occupational or speech and language therapy are made with the family’s consent. All children under 5 undergo a face-to-face physical examination with a pediatrician, where blood and genetic testing may be considered.

### CHATA Index Assessment Procedures

The CHATA index pathway, designed to be compatible with National Institute for Health and Care Excellence guidelines for autism assessment [18] and to replace, where appropriate, usual care CHAND assessment step 4 above, involves 2 steps:

1. Digital questionnaires: participants complete a series of self-administered questionnaires within 45 minutes on an NHS-compatible digital platform.
2. Online clinical observation: this is followed by a real-time, semistructured parent-child interaction through video conferencing, taking between 30 and 60 minutes depending on the need for interpreting.

Both the questionnaire and observational data are hosted on Drupal, a secure, open-source web application, locally hosted on an NHS server.

### Digital Questionnaires

The questionnaire was developed by integrating items that effectively identified autistic behaviors in preschool children from the following sources:

1. 3di interview, a computerized diagnostic tool used by UK Autism assessment teams [19]. The full 3di comprises nearly 200 questions, applicable across the full range of children and adolescent ages. We developed a preschool version using data from 1,437 children referred to the Great Ormond Street Hospital Social Communication Disorder Clinic (2005-2023). Using a discriminant function analysis, we identified 41 questions that best discriminated preschool children (n=356) assigned an autism diagnosis by the multidisciplinary clinic. From nearly 200 questions, 41 were selected for their effectiveness in distinguishing autism in preschool children. These interview questions were reconfigured into questionnaire format for CHATA.
2. The Modified Checklist for Autism in Toddlers, a screening tool for children under 30 months, consisting of 20 questions for parents [20].
3. The Developmental Check-In, a brief ASD screening tool validated for Hispanic children aged 24-60 months, includes 26 questions with accompanying photographs to aid parents with limited literacy [21,22].

An audit of the CHAND waiting list identified Urdu and Bengali (Sylheti and Dakar dialects) as the most frequently spoken languages after English. Questionnaires were professionally translated and back-translated, with parent focus groups assessing their accuracy and acceptability. Audio descriptions were created for participants with low literacy and uploaded alongside the written questionnaires.

Five parents/carers of children under 5 years of age who had undergone autism assessment participated in usability testing using the “think aloud approach” and the quantitative System Usability Scale (SUS; Multimedia Appendix 1), standard usability assessments [23,24]. Usability feedback was broadly positive, with critical feedback focused on the need to ensure clear instructions for accessing the questionnaires.

### Online Observations

The online observations will be conducted by an experienced clinician in preschool autism assessment, trained in the ADOS-2 autism assessment. Parents will use a broadband phone camera for the session, guided by the clinician through semistructured interactions with their child. This online observation was developed informed by the TELE-ASD-PEDS model, where the clinician remotely observes and instructs the caregiver in real-time tasks [25]. Scoring will align with the ADOS-2 methodology [16]. For the purposes of this pilot study sessions will be recorded for independent analysis to assess inter-rater reliability.

The assessment includes a 10- to 20-minute clinical interview, followed by a structured real-time observation. The clinician will review preassessment results to tailor the interview, focusing on specific concerns. During the observation, the child will perform activities with common household items and toys, guided by detailed instructions provided in advance. This will assess social communication, interaction, and restricted or repetitive behaviors. Interpreters will assist families who do not speak English as their first language, translating instructions and responses.

### Recruitment to Pilot Study

Families receiving health services at an inner London clinic, specifically those on the autism assessment waiting list, will be recruited. Potential participants will be contacted through telephone or email and provided with detailed study information. Participants can discuss any questions with the research assistant through various communication methods. Cultural advocates will ensure cultural sensitivity during recruitment (see inclusion and exclusion criteria in Textbox 1).

Cultural advocates and trained research team members will ensure a supportive environment.

**Inclusion and exclusion criteria.**

**Inclusion criteria**

- Parents and caregivers of children aged 5 or younger on the autism assessment waiting list in Newham, East London.
- Residents of Newham.
- Parents and caregivers aged 18 or older.

**Exclusion criteria**

- Inability to give consent.
- Known safeguarding concerns.
- Nonspeakers of English, Urdu, or Bengali.

#### Sample Size and Sample Size Justification

We will recruit 60 families for the main pilot study (including the usability testing) and 10-15 participants for the qualitative sub-study.

For clinical validation with approximately 80% power and an estimated 90% prevalence, a minimum sample size of 34 is needed to detect high sensitivity [26]. To detect high specificity with similar power, a minimum of 310 is required. A prestudy audit of the CHAND service showed a 90% autism diagnosis rate, like the 86% conversion rate in Bradford Autism Services [9]. Given the pilot nature of this study, we chose a pragmatic sample size of 50-60, prioritizing sensitivity assessment over specificity.

The sample size for quantitative usability testing is driven by the overall pilot sample size.

Approximately 15 participants are anticipated for the qualitative study, sufficient to reach thematic saturation and thoroughly explore core themes and patterns.

#### Estimated Diagnostic Accuracy

Families will complete both parts of the CHATA assessment, the online questionnaire and a structured videoconferencing clinical assessment, before their usual face-to-face CHAND clinical assessment. Following the CHATA assessment, children will proceed with the standard clinical assessment, including an in-person physical examination by a pediatrician and review at the MDT meeting for complex cases. This process (from the first CHATA assessment to the initial CHAND assessment) is expected to be finished within 6 weeks and will not affect their position on the clinical service waiting list.

Families will receive general autism information but no diagnosis from the CHATA assessment. The CHATA assessment results will be classified as autistic, nonautistic, or unsure by the research clinician but will not be shared with families or the clinical team to maintain blinding. The CHAND clinical team will record their own assessment outcomes and disclose them to the family as per usual processes. An independent researcher will compare the CHATA diagnostic outcome with the CHAND diagnostic outcomes from electronic health records to determine sensitivity and specificity.

#### Quantitative Usability

All families enrolled in the pilot will complete the SUS after completing the online questionnaires [24]. The SUS is a validated, widely used tool for measuring the usability of systems, with scores ranging from 0 to 100. The SUS consists of 10 items, with responses recorded on a 5-point Likert scale ranging from “Strongly Disagree” to “Strongly Agree.” Participants’ responses will be anonymized and collected into a database for analysis.

#### Qualitative Acceptability, Feasibility, and Usability Substudy

A qualitative study will include 10-15 families from the main study, chosen to represent diverse languages and experiences. This subset will help assess the acceptability and feasibility of the CHATA process, focusing on both positive aspects and areas for improvement. Recruitment will end when thematic saturation is reached, meaning no new themes emerge from the data. Data analysis will continue until no new codes appear, only recurring themes. In addition, 2-5 health care providers involved in autism assessments will be interviewed to gather their perspectives on the CHATA assessment, including its strengths and challenges. All interviews will be recorded with the participants’ consent.

#### Time Taken for Assessment

Informed by a recent study estimating the cost taken to undertake autism assessments in UK NHS services [27], we will estimate the time taken for each child to be seen under the usual care pathway, recording the grade of health care providers undertaking the assessments and compare that to the time taken to be seen under CHATA. We will then estimate costs associated with these using standard NHS outpatient costings.

### Quantitative Data Analysis

#### Usability Data

The SUS score for each participant will be calculated using the standard method: for each of the 10 items, scores range from 0 to 4. Positive items (1,3,5,7,9) will have 1 subtracted from the response, while negative items (2,4,6,8,10) will have the response subtracted from 5. The total score will be multiplied by 2.5, resulting in a final SUS score ranging from 0 to 100.

To summarize the CHATA online questionnaire usability, we will compute the mean, median, and SD of SUS scores. Scores will be interpreted as follows: above 68 is considered above average, 50 to 68 is marginally acceptable, and below 50 may indicate usability issues. We will perform subgroup analyses to compare SUS scores across different demographics (eg, age, language, and education) using *t* tests or ANOVA, as appropriate. The mean SUS score, with a 95% CI, will provide a precise estimate of usability, and significant differences between groups will be reported. If the mean SUS score falls below the acceptable threshold of 68, we will conduct qualitative follow-up interviews to explore specific usability issues. The findings will inform any necessary revisions to improve the CHATA pathway’s usability.

#### Diagnostic Accuracy

To evaluate the diagnostic accuracy of the CHATA pathway, we will calculate sensitivity, specificity, and positive predictive value (PPV; Multimedia Appendix 2). Sensitivity measures the pathway’s ability to correctly identify autism, calculated as the number of true positives divided by the sum of true positives and false negatives. Specificity, reflecting the pathway’s ability to correctly identify nonautistic cases, is calculated as the number of true negatives divided by the sum of true negatives and false positives; however, we may not have sufficient power to calculate this. PPV, which indicates the likelihood that a positive result truly indicates autism, is determined by dividing the number of true positives by the total number of positive results. These metrics will be assessed using data from the pilot cohort to evaluate the CHATA pathway’s accuracy in diagnosing autism in young children from diverse backgrounds.

#### Interrater Reliability

A second clinician will review about 10% of the online pilot assessment recordings and compare their diagnostic decisions with those of the original clinician. For inter-rater reliability, we will analyze the consistency of ratings across independent reviewers who are blinded to the initial results. Out of 13 observations, randomly selected from 60, will be reviewed independently by a second clinician and supervised by a third senior clinician. All reviewers will be trained to ensure consistent understanding of the assessment criteria. The primary measure of inter-rater reliability will be Cohen κ for categorical data (diagnosis present or absent), with values indicating the level of agreement (eg, <0 as no agreement, 0.61-0.80 as substantial agreement). The analysis will determine the consistency of the CHATA pathway across different clinicians, with high reliability indicating reliable use of the pathway and lower reliability suggesting areas for improvement. Results will be reported with CIs, and significant discrepancies will prompt a follow-up review to address potential issues in training, criteria, or guidance.

#### Qualitative Analysis

We will transcribe and, if necessary, translate the interview data for analysis using reflexive thematic analysis with an inductive approach. This analysis will evaluate the acceptability, feasibility, usability, and barriers or enablers of the assessment tools. A feasibility study will gather insights from parents, caregivers, and clinicians on both online and in-person assessments to identify effective aspects and challenges.

Interviews will be conducted within 2 weeks of the online assessment, either in-person at Child Development Clinics or online based on participants’ preferences. The interviews will be broad to capture diverse experiences, with topic guides (Multimedia Appendix 3) adjusted as new themes emerge. Qualitative data will be analyzed using NVivo [Lumivero] software, guided by the Theoretical Framework of Acceptability and the Theoretical Domains Framework [28,29]. The Theoretical Framework of Acceptability examines acceptability across 7 domains, including affective attitude and perceived effectiveness. The Theoretical Domains Framework integrates 33 behavior change theories and 14 domains to explore factors influencing behavior change, helping to identify barriers and enablers to implementation. We will use thematic analysis as outlined by Braun and Clarke [30], a widely used method for identifying, analyzing, and interpreting patterns of meaning within data. This approach is flexible and provides a detailed, nuanced understanding of the data, making it well-suited for the exploratory nature of this study. Since the study is qualitative and does not start with a pre-existing hypothesis, thematic analysis is particularly appropriate for uncovering insights.

The analysis will be reflexive, meaning it will involve continuous reflection and iteration by the research team. Out of 2 team members will independently familiarize themselves with the data, then regularly meet to discuss themes, resolve discrepancies, and finalize the themes and subthemes. The findings from this analysis will inform further iterations and refinements of the assessment tools to ensure they are as acceptable and effective as possible for both providers and recipients of the assessments.

### Ethical Considerations

This study has been approved by the London Bloomsbury Research Ethics Committee (reference 22/LO/0751).

Measures to support participants include clear communication, confidentiality assurance, flexible scheduling, addressing recruitment barriers, providing interpreters, offering supporting letters for nursery absences, and incentives like vouchers.

Children flagged for concerns related to Adverse Childhood Experiences or safeguarding will be assessed using the standard pathway, following all ELFT safeguarding protocols.

Before participation, each participant will review the information sheet (Multimedia Appendix 4) and consent form with a research team member, who will address any questions. Consent can be provided in various formats: face-to-face, by post, email, video call, or telephone. Participants will indicate their preferred communication method and availability. Materials and consent forms will be translated into participants’ preferred languages if needed. Non-English speakers will have the option to discuss the study with an interpreter through phone. Before the observation assessment, participants will receive detailed instructions and consent to record the session if desired. Researchers will be trained in consent processes, including assessing mental capacity. Parents will assess their child’s willingness to participate; if a child shows discomfort or resistance, they will not be enrolled in the study.

Data will be anonymized once data collection is completed and before any analysis. Digital recordings from qualitative interviews will be securely transferred to a dedicated NHS server drive and permanently deleted from recording devices and video conferencing platforms once verified. Pseudonymized transcripts will be stored on this secure drive, separate from the original recordings, which will be kept in a password-protected folder. Participants’ contact details will be stored in a separate, password-protected database on NHS servers. Hard copies of pseudonymized transcripts and research notes may be printed and kept in locked filing cabinets. Anonymized data will be retained on secure servers for 10 years before permanent deletion. All data handling will comply with the Data Protection Act 2018, with the participating NHS Trust as the Data Controller and Chief Investigators as data custodians.

## Results

Chata project funding commenced in April 2021, ethics approval obtained in June 2022 pilot study data collection commenced in April 2023, and completed in Oct 2024. Pilot study end date is March 2025. As of Nov 2024, we had enrolled 57 participants in the pilot study and 12 in the qualitative substudy.

## Discussion

### Overview

This paper outlines our protocol for refining and evaluating a novel online autism assessment system tailored for children under 5 within East London’s culturally and linguistically diverse population. The primary goal is to address the limitations of traditional autism assessments, which include high costs, lengthy procedures, and extensive waiting lists that often exceed 2 years, as well as reduced applicability to non-White and non–English-speaking groups. By the end of this study, we anticipate being able to describe the sensitivity, specificity, quantitative and qualitative usability, interrater reliability, acceptability, and feasibility of the CHATA pathway.

### Feasibility and Strategies

Conducting this trial presents inherent challenges due to the complexity of autism assessments in young children and the diverse needs of our target population. We are focusing on developing tools that are effective across diverse cultural and linguistic backgrounds. By integrating feedback from PPI and using non–English languagebased methods, we aim to ensure that the tools are culturally and linguistically appropriate. This approach is supported by previous research emphasizing the importance of cultural sensitivity in diagnostic tools [31,32]. To maximize the use of available clinical time, we are collecting parental information online before the assessment and ensuring that direct observations are concise. Literature supports this approach, noting that preassessment data collection and streamlined observations can significantly improve diagnostic efficiency [33].

We recognize the potential risks associated with implementing a novel assessment tool, including issues with engagement and the accuracy of online assessments. To mitigate these risks, we have designed a comprehensive evaluation process that includes both quantitative and qualitative assessments. This involves calculating sensitivity, specificity, and PPV to ensure diagnostic accuracy and conducting interrater reliability checks to maintain consistency [16,25].

We are taking several steps to minimize risks. All clinicians involved in the assessment process will undergo thorough training to ensure consistent application of the assessment criteria. This is critical for maintaining the reliability and validity of the assessments, as highlighted in previous research [33]. We have established robust protocols for data handling to ensure confidentiality and compliance with the Data Protection Act 2018. This includes secure storage of digital recordings and pseudonymized transcripts, as well as safeguarding participants’ contact details. Adherence to these protocols is essential for protecting participant privacy and maintaining trust [34]. The study will incorporate iterative feedback from participants and clinicians to continuously refine the assessment tools. This iterative approach, supported by research on diagnostic tool development, emphasizes the importance of ongoing evaluation, and adjustment to effectively meet user needs [35].

### Conclusion

Our study aims to develop a novel online autism assessment tool that addresses the limitations of traditional methods while being adaptable to diverse populations. By incorporating a range of assessment methods, ensuring rigorous training, and adhering to data protection standards, we strive to overcome the inherent challenges of this trial. Although the study is not designed to assess the cost-effectiveness of the assessment, it will focus on refining the tools and evaluation methods. Next steps include optimizing the efficiency of the questionnaire by reducing the redundancy of items and including visual content that has been created with ethnically diverse populations. Future research will need to assess sensitivity and specificity in a larger sample, address the long-term impact and cost-effectiveness of the online assessment system, and evaluate its applicability to a broader population, including those with lower digital literacy. The findings from this study will contribute valuable insights into autism diagnostics and provide a foundation for future trials aimed at improving assessment practices and reducing waiting times for autism services nationwide.

## Appendix

Multimedia Appendix 1 System Usability Scale.

Multimedia Appendix 2 Calculator for sensitivity, specificity, and positive predictive value (PPV).

Multimedia Appendix 3 Topic guides for parents and clinicians.

Multimedia Appendix 4 Information sheets.

## Abbreviations

ADOS-2: Autism Diagnostic Observation Schedule, Second Edition
ASD: autism spectrum disorder
CHAND: Children with Autism in Newham – Diagnosis Service
CHATA: Children with Autism Technology Enabled Assessment
LBN: London Borough of Newham
MDT: multidisciplinary team
NHS: National Health Service
PPI: patient and public involvement
PPV: positive predictive value
SUS: System Usability Scale

## References

[1] Salari N, Rasoulpoor S, Rasoulpoor S, Shohaimi S, Jafarpour S, Abdoli N, Khaledi-Paveh B, Mohammadi M (2022). The global prevalence of autism spectrum disorder: a comprehensive systematic review and meta-analysis. Ital J Pediatr.

[2] O'Nions E, Petersen I, Buckman JEJ, Charlton R, Cooper C, Corbett A, Happé F, Manthorpe J, Richards M, Saunders R, Zanker C, Mandy W, Stott J (2023). Autism in England: assessing underdiagnosis in a population-based cohort study of prospectively collected primary care data. Lancet Reg Health Eur.

[3] (2024). NHS Digital. Autism waiting time statistics.

[4] Community services dataset. NHS Digital.

[5] Zwaigenbaum L, Bauman ML, Choueiri R, Kasari C, Carter A, Granpeesheh D, Mailloux Z, Smith Roley S, Wagner S, Fein D, Pierce K, Buie T, Davis PA, Newschaffer C, Robins D, Wetherby A, Stone WL, Yirmiya N, Estes A, Hansen RL, McPartland JC, Natowicz MR (2015). Early intervention for children with autism spectrum disorder under 3 years of age: recommendations for practice and research. Pediatrics.

[6] Gordon-Lipkin E, Foster J, Peacock G (2016). Whittling down the wait time: exploring models to minimize the delay from initial concern to diagnosis and treatment of autism spectrum disorder. Pediatr Clin North Am.

[7] van 't Hof M, Tisseur C, van Berckelear-Onnes I, van Nieuwenhuyzen A, Daniels AM, Deen M, Hoek HW, Ester WA (2021). Age at autism spectrum disorder diagnosis: a systematic review and meta-analysis from 2012 to 2019. Autism.

[8] An evidence-based plan for addressing the autism assessmentsupport crisis (2024). An evidence-based plan for addressing the autism assessment and support crisi. Child of the North.

[9] (2024). Connected Bradford.

[10] Spain D, Stewart GR, Mason D, Milner V, Fairhurst B, Robinson J, Gillan N, Ensum I, Stark E, Happe F (2022). Telehealth autism diagnostic assessments with children, young people, and adults: qualitative interview study with England-wide multidisciplinary health professionals. JMIR Ment Health.

[11] Liu M, Ma Z (2022). Correction: a systematic review of telehealth screening, assessment, and diagnosis of autism spectrum disorder. Child Adolesc Psychiatry Ment Health.

[12] Katakis P, Estrin GL, Wolstencroft J, Sayani S, Buckley E, Mirzaei V, Heys M, Skuse D (2023). Diagnostic assessment of autism in children using telehealth in a global context: a systematic review. Rev J Autism Dev Disord.

[13] Stavropoulos KK, Bolourian Y, Blacher J (2022). A scoping review of telehealth diagnosis of autism spectrum disorder. PLoS One.

[14] Kryszak EM, Albright CM, Fell LA, Butter EM, Kuhlthau KA (2022). Clinician perspectives on telehealth assessment of autism spectrum disorder during the COVID-19 pandemic. J Autism Dev Disord.

[15] Baum F, MacDougall C, Smith D (2006). Participatory action research. J Epidemiol Community Health.

[16] McCrimmon A, Rostad K (2013). Test review: autism diagnostic observation schedule, second edition (ADOS-2) manual (Part II): toddler module. Journal of Psychoeducational Assessment.

[17] Billstedt E, Gillberg IC, Gillberg C (2007). Autism in adults: symptom patterns and early childhood predictors. Use of the DISCO in a community sample followed from childhood. J Child Psychol Psychiatry.

[18] (2017). Autism spectrum disorder in under 19s: recognition, referral and diagnosis. National Institute for Health and Care Excellence.

[19] Skuse D, Warrington R, Bishop D, Chowdhury U, Lau J, Mandy W, Place M (2004). The developmental, dimensional and diagnostic interview (3di): a novel computerized assessment for autism spectrum disorders. J Am Acad Child Adolesc Psychiatry.

[20] Robins DL, Casagrande K, Barton M, Chen CA, Dumont-Mathieu T, Fein D (2014). Validation of the modified checklist for Autism in toddlers, revised with follow-up (M-CHAT-R/F). Pediatrics.

[21] Harris JF, Coffield CN, Janvier YM, Mandell D, Cidav Z (2021). Validation of the developmental check-in tool for low-literacy autism screening. Pediatrics.

[22] Janvier YM, Coffield CN, Harris JF, Mandell DS, Cidav Z (2019). The developmental check-in: development and initial testing of an autism screening tool targeting young children from underserved communities. Autism.

[23] Alhadreti O, Mayhew P (2017). To intervene or not to intervene: an investigation of three think-aloud protocols in usability testing. J Usability Studies.

[24] Brooke J, Jordan PW, Thomas B, McClelland IL, Weerdmeester B (1996). SUS: a quick and dirty usability scale. Usability Evaluation in Industry.

[25] Wagner L, Corona LL, Weitlauf AS, Marsh KL, Berman AF, Broderick NA, Francis S, Hine J, Nicholson A, Stone C, Warren Z (2021). Use of the TELE-ASD-PEDS for autism evaluations in response to COVID-19: preliminary outcomes and clinician acceptability. J Autism Dev Disord.

[26] Bujang MA, Adnan TH (2016). Requirements for minimum sample size for sensitivity and specificity analysis. J Clin Diagn Res.

[27] Male I, Farr W, Bremner S, Gage H, Williams P, Gowling E, Honey E, Gain A, Parr J (2023). An observational study of individual child journeys through autism diagnostic pathways, and associated costs, in the UK national health service. Front Rehabil Sci.

[28] Sekhon M, Cartwright M, Francis JJ (2017). Acceptability of healthcare interventions: an overview of reviews and development of a theoretical framework. BMC Health Serv Res.

[29] Atkins L, Francis J, Islam R, O'Connor D, Patey A, Ivers N, Foy R, Duncan EM, Colquhoun H, Grimshaw JM, Lawton R, Michie S (2017). A guide to using the theoretical domains framework of behaviour change to investigate implementation problems. Implement Sci.

[30] Clarke V, Braun V (2016). Thematic analysis. The Journal of Positive Psychology.

[31] Kapp SK, Gillespie-Lynch K, Sherman LE, Hutman T (2013). Deficit, difference, or both? Autism and neurodiversity. Dev Psychol.

[32] Heys M, Gibbons F, Haworth E, Medeiros E, Tumbahangphe KM, Wickenden M, Shrestha M, Costello A, Manandhar D, Pellicano E (2018). The estimated prevalence of autism in school-aged children living in rural Nepal using a population-based screening tool. J Autism Dev Disord.

[33] Gotham K, Pickles A, Lord C (2009). Standardizing ADOS scores for a measure of severity in autism spectrum disorders. J Autism Dev Disord.

[34] Howe N, Giles E, Newbury-Birch D, McColl E (2018). Systematic review of participants' attitudes towards data sharing: a thematic synthesis. J Health Serv Res Policy.

[35] Dopp AR, Parisi KE, Munson SA, Lyon AR (2020). Aligning implementation and user-centered design strategies to enhance the impact of health services: results from a concept mapping study. Implement Sci Commun.

