# Supplementary material for: Novel Procedures for Evaluating Autism Online in a Culturally Diverse Population of Children: Protocol for a Mixed Methods Pathway Development Study
Source: JMIR Res Protoc. 2025 Feb 11;14:e55741. doi: 10.2196/55741 (PMC11862771; doi:10.2196/55741)
Supplement: Multimedia Appendix 1 [file resprot_v14i1e55741_app1.docx]

System Usability Scale

Strongly Strongly

Disagree Agree

1. I think that I would like to

use this system frequently

2. I found the system unnecessarily
 complex

1 2 3 4 5

1 2 3 4 5

3. I thought the system was easy
to use

1 2 3 4 5

4. I think that I would need the

support of a technical person to
be able to use this system

1 2 3 4 5

5. I found the various functions in
this system were well integrated

1 2 3 4 5

6. I thought there was too much
inconsistency in this system

1 2 3 4 5

7. I would imagine that most people
would learn to use this system

very quickly 1 2 3 4 5

8. I found the system very cumbersome to use

1 2 3 4 5

9. I felt very confident using the

System

1 2 3 4 5

10. I needed to learn a lot of

things before I could get going

with this system 1 2 3 4 5

Brooke J. SUS: a quick and dirty usability scale. In: Jordan PW, Thomas B, McClelland IL, Weerdmeester B, editors. Usability Evaluation in Industry. Milton Park, in Oxfordshire. Taylor & Francis; 1996:4-7.

[View publication stats](https://www.researchgate.net/publication/228593520)
