# Supplementary figures and images for: Novel Procedures for Evaluating Autism Online in a Culturally Diverse Population of Children: Protocol for a Mixed Methods Pathway Development Study
Source: JMIR Res Protoc. 2025 Feb 11;14:e55741. doi: 10.2196/55741 (PMC11862771; doi:10.2196/55741)

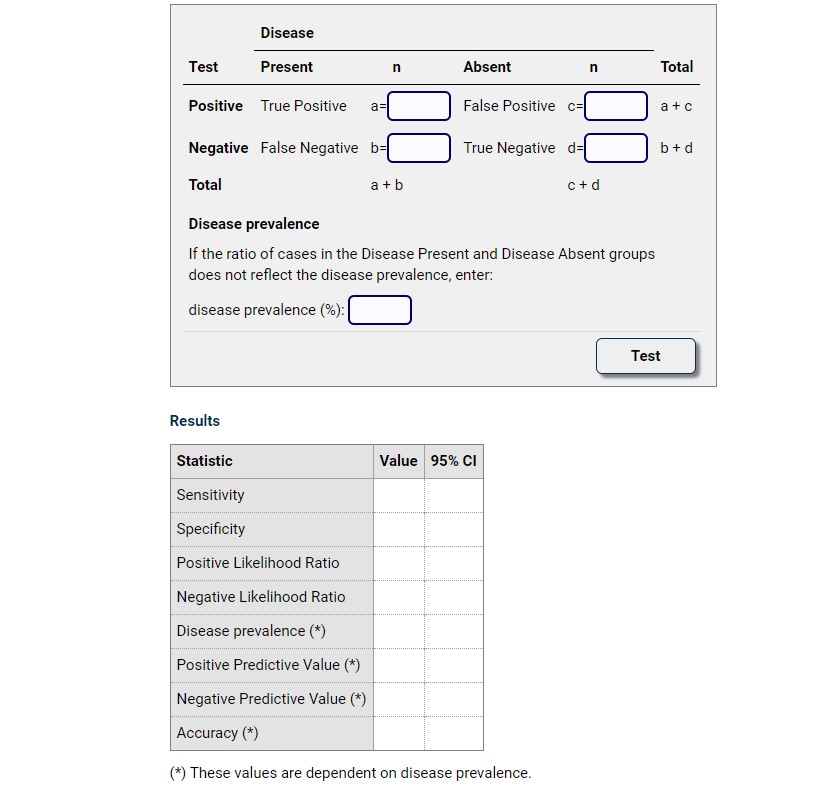


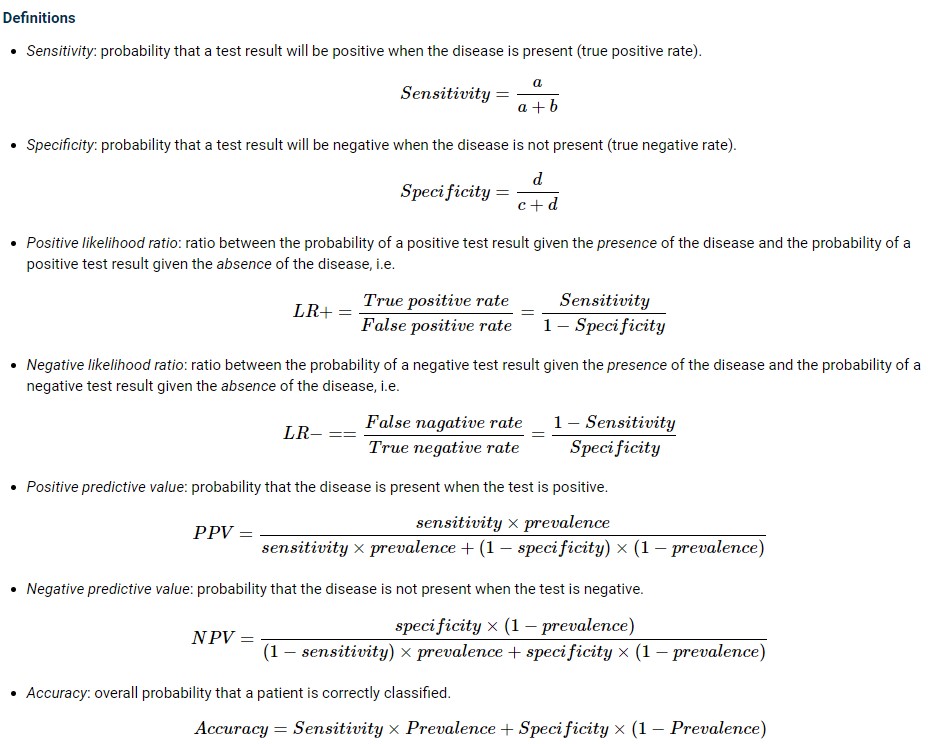

Supplement: Multimedia Appendix 2 [file resprot_v14i1e55741_app2.docx]
