# Supplementary material for: Novel Procedures for Evaluating Autism Online in a Culturally Diverse Population of Children: Protocol for a Mixed Methods Pathway Development Study
Source: JMIR Res Protoc. 2025 Feb 11;14:e55741. doi: 10.2196/55741 (PMC11862771; doi:10.2196/55741)
Supplement: Multimedia Appendix 3 [file resprot_v14i1e55741_app3.pdf]

NHS East London NHS Foundation Trust

IMPROVING AUTISM ASSESSMENTS CHATA CHILDREN'S AUTISM TECHNOLOGY-ASSISTED ASSESSMENTS

TOPIC GUIDE 2: Clinician Feasibility & Acceptability Guide (pilot phase)

Introduction

Hello, thank you very much for taking the time to speak to me today. My name is _____ and I work for the Specialist Children's and Young People's Service in the East London Foundation Trust. Our service provides care for children, adolescents, and families in Newham, and aims at improving the quality of life of all the people we serve. The interview will take around 20-40 minutes.

This interview relates to a study that is evaluating online autism assessments.

Before we start, can I check whether you have:

- Had an information sheet
- Signed a consent form
- Agreed to audio recording of the discussion

Before going further, do you have any questions about the purpose of the study, or the documents you have been given - for example the participant information sheet or consent form? Is there anything that isn't clear?

The purpose of this interview is to get your perspective and views on online autism assessment.

To give you a brief overview, this project is called CHATA, which stands for Children's Autism Technology-Assisted Assessments (**CHATA)**, and what we're doing is trying to design a way to conduct autism assessments online or partially online.

We're doing this for many reasons.

First, during COVID we were forced to do some of our developmental assessments online – including those for autism. We learned a lot about what might be possible. And we want to know what parts of the autism assessment, if any, we might want to continue to do online.

Second, referrals for autism assessments are rising faster than we can realistically manage within our current resources. So, we need to come up with ways of working smarter. This project looks about better and more efficient ways of assessing autism.

Third, we need to learn from the children and families we care for how to be better. This is only possible through collecting information about children's development and their experience of our service. This project will collect this information.

Finally, we work with a diverse community and many of the autism tools we use have been developed with very specific types of children (generally white, English-speaking American children). Therefore, we will work hard to make sure the tools we use are suitable for use irrespective of ethnicity and language. Your opinions are very important to us and will help us make the assessments more suitable for parents/carers and the children involved. We expect different

opinions, and there are no right or wrong answers. All comments are welcome, as we just want your honest views.

If there are any questions you do not wish to answer, that's not a problem. If you need a break or wish to stop the interview, that's fine, please just let us know.

Affect Attitude (TFA) & Acceptability (Feasibility framework) - Initial Impressions

How do you feel about the online assessment?

Was there anything about it that was good or bad,
aspects that you did or didn't like?

Intervention Coherence (TFA)

What do you think the online assessment was trying to achieve?

Beliefs about consequences (TDA)

What do you think the benefits or positive aspects
of the online assessment are?

What do you think the drawbacks or negative aspects
of the online assessment are?

Overall, do you think the benefits outweigh the
negative aspects of the online assessment? And why?

Demand (Feasibility framework)

How do you feel about assessing children for autism using online methods?

If you were advising a family about accessing an online autism assessment now
or an in-person autism assessment in several months' time, how would you approach that?

In your view, how much need is there for online assessments?

Perceived Effectiveness (TFA)

Do you think online autism assessment can assess a child
for autism accurately?

Do you think online autism assessment can improve
the quality of care for children?

Integration (Feasibility framework) – contextual

Can you think of any ways that the format and delivery of online autism
assessment may or may not be suitable in Newham? And the broader UK?

As a clinician what might affect your decision to offer an online autism assessment to a family?

How do you think others in your professional community would view online autism assessment?

Ethicality (TFA)

Do you feel parents/carers would feel safe sharing their feelings/experiences in an online setting? On questionnaires and/or online appointments.

Do you think any aspect of the online assessment could be offensive/unacceptable to you or parents, or make people feel uncomfortable?

Do you think this online assessment will improve or worsen some of the inequalities among children and their families?

Burden (TFA)

Do you think taking part in online assessment will give you extra work or affect other parts of your life in any way?

Is there anything about completing online questionnaires or online appointments that concerns you?

Opportunity Cost (TFA)

What kind of things do you think clinicians would have to give up, to engage with this programme?

Self-efficacy (TFA & TDF)

How confident would you feel assessing a child for autism online?

What is easy/not easy about online assessment?

Do you think online assessment can empower clinicians in any way?

Adaptation (feasibility framework) – contextual factors

Is there anything we could add or change about the online assessment to make it more suitable for clinicians?

Are there more effective or suitable ways of assessing autism?

Practicality (Feasibility framework)

How can this online assessment be provided in a way that is acceptable and accessible?

Did you find the online assessment easy to use and understand? And if not, why not?

Would you like any changes to be made to the online autism assessment?

Any other questions or comments?

TOPIC GUIDE 1 Parent Feasibility & Acceptability Guide (pilot phase)

Introduction

Hello, thank you very much for taking the time to speak to me today. My name is _____ and I work for the Specialist Children's and Young People's Service in the East London Foundation Trust. Our service provides care for children, adolescents, and families in Newham, and aims at improving the quality of life of all the people we serve. The interview will take around 20-40 minutes.

This interview is a part of a study aiming to improve how we assess children and young people for possible autism.

Before we start, can I check whether you have:

- Had an information sheet
- Signed a consent form
- Agreed to audio recording of the discussion

Before going further, do you have any questions about what the study is about or why we are doing it, or the documents you have been given - for example the participant information sheet or consent form? Is there anything that isn't clear?

The purpose of this interview is to listen to your views on online autism assessment. For example, we want to know how you would feel using this online service for your child's autism assessment We're interested in hearing about the patient experience so we can make the option of an online assessment as accessible for families as possible.

I will give you some information about the project. This project is called CHATA, which stands for Children's Autism Technology-Assisted Assessments (**CHATA)**. What we're doing is trying to design a way to run autism assessments online or partially online.

We're doing this for many reasons.

First, during COVID we were forced to do some of our developmental assessments online – including those for autism. We learned a lot about what might be possible. And we want to know what parts of the autism assessment, if any, we might want to continue to do online.

Second, referrals for autism assessments are rising faster than we can realistically manage within our current resources. So, we need to come up with ways of working smarter. This project looks at better and more efficient ways of assessing autism. The data shows that the earlier assessments are completed, the sooner interventions can be accessed and families can get the support they need to enable their children to receive tailored support.

Third, we need to learn from the children and families we care for how to be better. This is only possible through collecting information about children's development and their experience of our service. This project will collect this information.

Finally, we work with a diverse community and many of the autism tools we use have been developed with very specific types of children (generally white, English-speaking American children).

Therefore, we will work hard to make sure the tools we use are suitable for use irrespective of ethnicity and language. Your opinions are very important to us and will help us make the assessments more suitable for parents/carers and the children involved. We expect different opinions, and there are no right or wrong answers. All comments are welcome, as we just want your honest views.

Nothing said during this interview will adversely affect the care you or your child receives.

If there are any questions you do not wish to answer, that's not a problem. If you need a break or wish to stop the interview, that's fine, please just let us know.

If you have any questions or complaints about the research study, you can contact Dr Michelle Heys (m.heys@ucl.ac.uk) or Dr Shermina Sayani (shermina.sayani@nhs.net) at any time. If you feel that your complaint has not been handled to your satisfaction, please contact the Patient Advice and Liaison Service (PALS), which can help you sort out any problems or issues you may have with a Trust service and enable you to make decisions about your care and treatment.

The PALS team can be contacted via FREEPHONE 0800 783 4839 or Email: elft.pals@nhs.net.

Affect Attitude (TFA) & Acceptability (Feasibility framework) - Initial Impressions

How did you feel about doing the online assessment?

Was there anything about it that was good or bad, things that you did or didn't like?

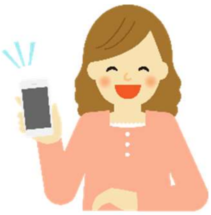

Intervention Coherence (TFA)

What do you think the online assessment was trying to achieve?

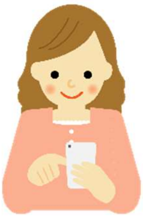

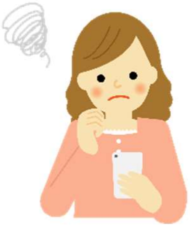

Beliefs about consequences (TDA)

What do you think the benefits or positives of the online assessment are?

What do you think the drawbacks or negatives of the online assessment are?

Overall, do you think there are more positive things or negative things about an online assessment? And why?

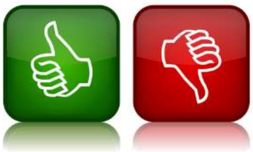

Demand (Feasibility framework)

How would you feel about having your child assessed for autism online in the way that you tried?

If you could choose between online autism assessment now or an in-person autism assessment in several months' time, which would you choose and why?

In your opinion, do we need online assessments?

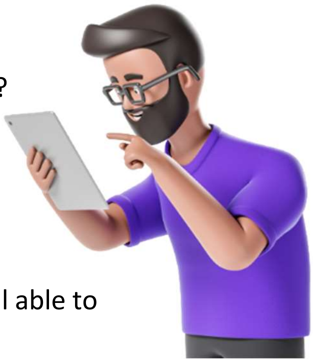

Perceived Effectiveness (TFA)

If your child received an online assessment confirming autism, would you trust it and feel able to move on and access interventions based on this alone?

Do you think online autism assessment affects care for children?

Integration (Feasibility framework) – contextual

Can you think of anything about the way the online autism assessment was delivered that may or may not be suitable in Newham? And more broadly, in the UK?

As a parent/carer what might affect your decision to take part in an online autism assessment for your child?

What would others (friends, family, education professionals, and health professionals) think of your decision to take part in an online assessment for autism?

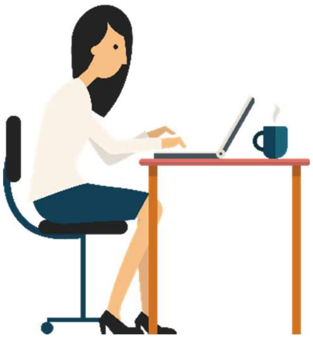

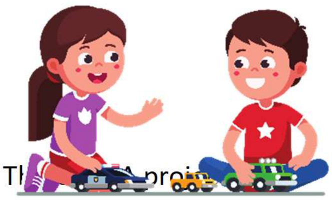

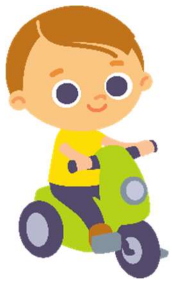 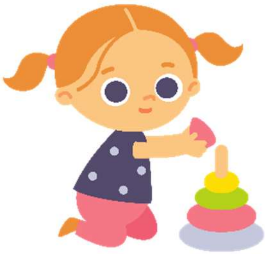 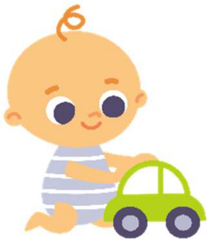 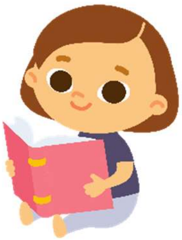 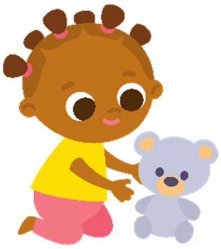

Ethicality (TFA)

Do you think parents/carers would feel safe sharing their feelings and experiences in an online questionnaire and/or appointment?

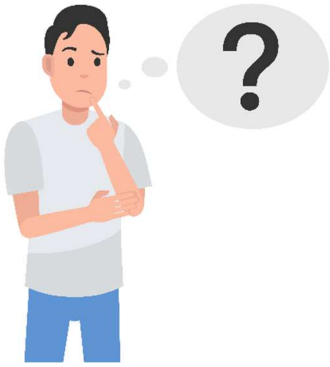

Is there anything about the online assessment that could be offensive/unacceptable to you or other parents, or make people feel uncomfortable?

Do you think an online assessment will affect some of the inequalities that children and their families face?

Burden (TFA)

Do you think taking part in online assessment will give you extra work?

Is there anything about completing online questionnaires or online appointments that concerns you?

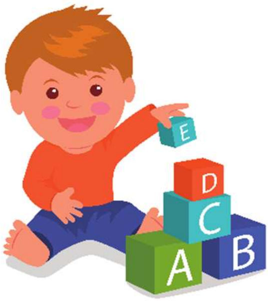

Do you think online assessment will affect other parts of your life in any way?

Opportunity Cost (TFA)

What kind of things do you think parents would have to give up to take part in online autism assessment for their child? And do you think it's worth it?

Self-efficacy (TFA & TDF)

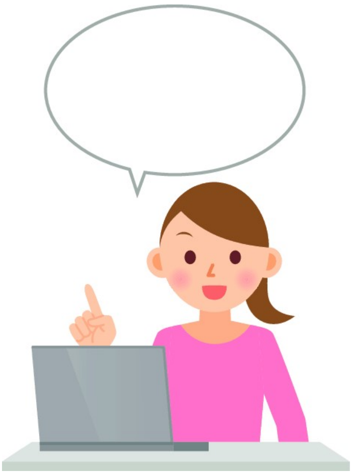

How confident would you feel taking part in an online autism assessment for your child?

What is easy/not easy about online assessment?

Do you think online assessment can empower/help parents/carers in any way?

Adaptation (feasibility framework) – contextual factors

Is there anything we could add or change about the online assessment to make it more suitable/better for parents/carers?

How else do you think we could get to know your child in order to assess whether or not they might be autistic?

Practicality (Feasibility framework)

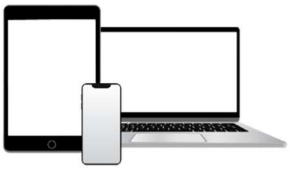

Do you have internet (connection) in your home? If not, why?

Which devices (e.g., computer, phone) do you use to connect to the internet?

Which device did you use to access the online autism assessment?

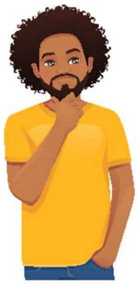

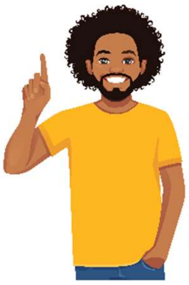

Did you find the online assessment easy to use and understand? And if not, why not?

Would you like any changes to be made to the online autism assessment?

Any other questions or comments?
