# Supplementary material for: Novel Procedures for Evaluating Autism Online in a Culturally Diverse Population of Children: Protocol for a Mixed Methods Pathway Development Study
Source: JMIR Res Protoc. 2025 Feb 11;14:e55741. doi: 10.2196/55741 (PMC11862771; doi:10.2196/55741)
Supplement: Multimedia Appendix 4 [file resprot_v14i1e55741_app4.pdf]

Participant Information Sheet

Parents/Carers

Pilot clinical validation of online assessment procedure

We are inviting you to take part in a research study taking place at the Specialist Children's and Young People's Service (SCYPS) in the East London Foundation Trust. Before you decide whether to take part, it is important that you know why we are doing the study and what is involved. Please read the following information carefully.

This research study is part of a project called CHATA, which stands for Children's Autism Technology-Assisted Assessments (CHATA). The CHATA project is funded by The Patrick Paul Foundation, the Sponsor Organisation (Sponsors number: H-2019) and the Data Controller is the East London Foundation Trust.

What we're doing is trying to design a way to run autism assessments online or partially online. We are using several existing autism screening and assessment measures alongside some novel tools and trialling them as part of autism assessments for children under 5 years old.

What is the purpose of the study?

The purpose of the study is to work with parents/carers and health professionals to evaluate whether online methods of screening and assessment for autism are feasible and acceptable to the ethnically and linguistically diverse community in Newham.

We aim to develop a validated, acceptable, efficient, and feasible method of assessing autism online that, when fully implemented, will be applicable to communities throughout the UK, irrespective of their ethnic diversity and which will be tailored to surmount language barriers.

Why have I been invited?

We are inviting parents and carers of a child who is being assessed for autism, who live in Newham and are 18 years or older to take part.

What would taking part involve?

If you decide to take part, we will invite you to complete a selection of online surveys in your own time (these will take approximately 30-45 minutes to complete), we will then invite you and your child to a 1-hour video appointment with a clinician where you will be asked to complete some play-based tasks with your child in front of the camera and also answer some questions about your child's development. We will then ask you to complete a short survey (10 minutes) and an interview with a researcher about how you found the process (20-40 minutes).

Will you record the appointment and interview?

Yes. We will ask for your consent (permission) to audio and/or video record the appointment and also the interview.

How much time will it take?

The total amount of time to complete the surveys, appointment, and follow-up survey and interview will be approximately 2-2.5 hours.

How many people are involved in this research?

We hope to involve around 35 parent/carers.

What happens if we cannot do face-to-face research due to social distancing or lockdown rules?

If we are unable to do face-to-face research, we will invite you to participate in a remote discussion or interview via phone or video call (e.g. Skype, Zoom, Microsoft Teams).

If you choose to have a video call, we will ask if you have access to a tablet, laptop, smartphone or PC. We will also check that you have internet access and that you can secure a private space at home. We will then email you an invitation and a link to join the discussion. If you require additional data to participate in the interview, we will reimburse you for these costs.

Do I have to take part in this research?

No. It is up to you to decide whether you would like to take part.

Will you reimburse my costs?

Yes, we will reimburse your travel costs. Also, you will receive either mobile data or a supermarket voucher as a token of appreciation for participating in the study.

What are the possible benefits of taking part?

You have the opportunity to aid the development of autism assessment tools and give your feedback on how we shape the online service.

What are the possible disadvantages of taking part?

A potential disadvantage is that you could become distressed talking about aspects of autism assessment. We will do our best to minimise any distress by creating a safe and supportive environment. A trained facilitator will be able to support you within the session and follow up afterwards for debrief.

You do not have to answer any questions if you do not feel comfortable. You can also leave the discussion at any point.

What if something goes wrong?

If you have any questions or complaints about the research study, you can contact Dr Michelle Heys (m.heys@ucl.ac.uk) or Dr Shermina Sayani (shermina.sayani@nhs.net) at any time. If you feel that your complaint has not been handled to your satisfaction, please contact the Patient Advice and Liaison Service (PALS), which can help you sort out any problems or issues you may have with a Trust service and enable you to make decisions about your care and treatment.

The PALS team can be contacted via FREEPHONE 0800 783 4839 or Email: elft.pals@nhs.net

How will we use information about you?

If you choose to enrol in this study, we will ask you for your contact details. This includes: your name, address, mobile number and email address. This will be kept confidential. Only the Study Coordinator will have access to your contact details. They will destroy this information at the end of the study.

Any audio and video recordings made will be kept securely. Only the research team and an external transcriber will have access to the recordings. The recordings will be destroyed at the end of the study.

A typed-up record of your answers (transcript) will be kept for a longer period. This will be stored anonymously. This means that your name, and any other identifying information, will not be linked to what you have said.

This anonymised information will be shared among members of the research team. We will also use this anonymous information in research reports and publications. In the future, it may also be shared with other researchers at other institutions.

We will destroy all copies of the anonymous study data after 10 years.

Lastly, we will send a letter to your GP (or the health professional that referred the child) to notify them about the purposes of the study and your participation.

Limits to Confidentiality

If you tell us anything in the interview or discussion that makes the Study Coordinator worry that either you or your family are at risk of harm, the Study Coordinator may have to inform the Chief Investigator (Dr Michelle Heys). If this happens, the Study Coordinator will discuss this with you, before they do this.

Will you have access to any medical records?

We will ask your consent (permission) to access the medical records of the child or young person in your care. This is so we can collect information about the care they have already received and to know what services they have been receiving in the last 12 months. This information will be anonymised and not linked to any identifying information (such as the child or young person's name or contact details).

What are your choices about how your information is used?

You can stop being part of the study at any time by letting the Study Coordinator know. You do not have to give a reason.

If you decide to withdraw, the Study Coordinator will ask what you would like us to do with your data. You can request to have your personal data (e.g. any records of your name and contact details) erased at any time. However, we will keep the research data that you have already given us (e.g. your responses in interviews and group discussions). This information will not be linked to your name or contact details. Your data will have a code number instead.

Where can you find out more about how your information is used?

You can find out more about how we use your information:

- Visiting www.hra.nhs.uk/information-about-patients/
- Viewing our leaflet available from www.hra.nhs.uk/patientdataandresearch

· By asking one of the research team (see Study Coordinator details)

· By sending an email to the ELFT Data Protection Officer, Chris Kitchener at elft.dpo@nhs.net

· By ringing the DPO Office on 020 7655 4000

What will happen to the findings of this study?

We will publish the findings of this research in reports and scientific journals. When we report what you have told us we will not use your name or any other personal identifying information about you. Instead, we will replace your name with a code (number). In this way, the information you give us will be anonymised.

We will also hold some events in Newham to share our findings with the wider community. We will invite you to attend and participate in these events.

Who is organising and funding this study?

The research is being organised by Dr Michelle Heys (Chief Investigator) who is a Consultant Community Paediatrician at the East London NHS Foundation Trust. The study is funded by the Patrick Paul Foundation.

How have patients and the public been involved in this study?

We asked a parent of a child who has undergone autism assessment to give their views on all the research documents (e.g. questionnaires and information sheets). We also asked for their advice and guidance on recruitment procedures. We have also held focus groups with parents of children who have undergone autism assessment with CHAND when designing the online assessment.

Who has reviewed this study?

This study has been independently peer-reviewed and has received regulatory approvals. The study has received HRA approval from the Health Research Authority (HRA) and Favourable Ethical Opinion from the Research Ethics Committee (REC)

Further information and contact details

If you would like to talk to someone about the study please contact:

Chief Investigator:

Dr Michelle Heys,

Email: michelle.heys@nhs.net

Consultant Paediatrician and Paediatric Lead for Autism Pathway:

Dr Shermina Sayani

Email: shermina.sayani@nhs.net

Participant Information Sheet

Testing usability of the online screening and assessment

We are inviting you to take part in a research study taking place at the Specialist Children's and Young People's Service (SCYPS) in the East London Foundation Trust. Before you decide whether to take part, it is important that you know why we are doing the study and what is involved. Please read the following information carefully.

This research study is part of a project called CHATA, which stands for Children's Autism Technology-Assisted Assessments (CHATA). The CHATA project is funded by The Patrick Paul Foundation, the Sponsor Organisation (Sponsors number: H-2019) and the Data Controller is the East London Foundation Trust.

What we're doing is trying to design a way to run autism assessments online or partially online. We are using several existing autism screening and assessment measures alongside some novel tools and trialling them as part of autism assessments for children under 5 years old.

What is the purpose of the study?

The purpose of the study is to work with parents/carers and health professionals to evaluate whether online methods of screening and assessment for autism are feasible and acceptable to the ethnically and linguistically diverse community in Newham.

We aim to develop a validated, acceptable, efficient, and feasible method of assessing autism online that, when fully implemented, will be applicable to communities throughout the UK, irrespective of their ethnic diversity and which will be tailored to surmount language barriers.

Why have I been invited?

We are inviting parents and carers of a child who has been assessed for autism, who live in Newham and are 18 years or older to take part.

What would taking part involve?

If you decide to take part, we will invite you to visit the West Ham Lane Health Centre in Newham to take part in a small group to test and give us feedback on a variety of computer-based surveys and activities that screen and assess for autism in children. While you are completing the surveys and activities you will be asked questions and for your opinion by two researchers who will be in the room with you.

Will you record the discussions and interviews?

Yes. We will ask for your consent (permission) to audio and/or video record all discussions and interviews.

How much time will it take?

The group discussion/workshop will take approximately 1-2 hours.

How many people are involved in this research?

We hope to involve around 5 parent/carers.

What happens if we cannot do face-to-face research due to social distancing or lockdown rules?

If we are unable to do face-to-face research, we will invite you to participate in a remote discussion or interview via phone or video call (e.g. Skype, Zoom, Microsoft Teams).

If you choose to have a video call, we will ask if you have access to a tablet, laptop, smartphone or PC. We will also check that you have internet access and that you can secure a private space at home. We will then email you an invitation and a link to join the discussion. If you require additional data to participate in the interview, we will reimburse you for these costs.

Do I have to take part in this research?

No. It is up to you to decide whether you would like to take part.

Will you reimburse my costs?

Yes, we will reimburse your travel costs. Also, you will receive either mobile data or a supermarket voucher as a token of appreciation for participating in the study.

What are the possible benefits of taking part?

You have the opportunity to aid the development of autism assessment tools and give your feedback on how we shape the online service.

What are the possible disadvantages of taking part?

A potential disadvantage is that you could become distressed talking about aspects of autism assessment. We will do our best to minimise any distress by creating a safe and supportive environment. A trained facilitator will be able to support you within the session and follow up afterwards for debrief.

You do not have to answer any questions if you do not feel comfortable. You can also leave the discussion at any point.

What if something goes wrong?

If you have any questions or complaints about the research study, you can contact Dr Michelle Heys (m.heys@ucl.ac.uk) or Dr Shermina Sayani (shermina.sayani@nhs.net) at any time. If you feel that your complaint has not been handled to your satisfaction, please contact the Patient Advice and Liaison Service (PALS), which can help you sort out any problems or issues you may have with a Trust service and enable you to make decisions about your care and treatment.

The PALS team can be contacted via FREEPHONE 0800 783 4839 or Email: elft.pals@nhs.net

How will we use information about you?

If you choose to enrol in this study, we will ask you for your contact details. This includes: your name, address, mobile number and email address. This will be kept confidential. Only the Study Coordinator will have access to your contact details. They will destroy this information at the end of the study.

Any audio and video recordings made will be kept securely. Only the research team and an external transcriber will have access to the recordings. The recordings will be destroyed at the end of the study.

A typed-up record of your answers (transcript) will be kept for a longer period. This will be stored anonymously. This means that your name, and any other identifying information, will not be linked to what you have said.

This anonymised information will be shared among members of the research team. We will also use this anonymous information in research reports and publications. In the future, it may also be shared with other researchers at other institutions.

We will destroy all copies of the anonymous study data after 10 years.

Limits to Confidentiality

If you tell us anything in the interview or discussion that makes the Study Coordinator worry that either you or your family are at risk of harm, the Study Coordinator may have to inform the Chief Investigator (Dr Michelle Heys). If this happens, the Study Coordinator will discuss this with you before they do this.

If you choose to take part in the focus groups, we will not be able to ensure confidentiality during the focus groups. We will ask everyone taking part in the group to sign a confidentiality form agreeing not to share our conversation with anyone after the group. However, we cannot guarantee that this will not occur.

Will you have access to any medical records?

We will ask your consent (permission) to access the medical records of the child or young person in your care. This is so we can collect information about their original assessment and to know what services they have been receiving in the last 12 months. This information will be anonymised and not linked to any identifying information (such as the child or young person's name or contact details).

What are your choices about how your information is used?

You can stop being part of the study at any time by letting the Study Coordinator know. You do not have to give a reason.

If you decide to withdraw, the Study Coordinator will ask what you would like us to do with your data. You can request to have your personal data (e.g. any records of your name and contact details) erased at any time. However, we will keep the research data that you have already given us (e.g. your responses in interviews and group discussions). This information will not be linked to your name or contact details. Your data will have a code number instead.

Where can you find out more about how your information is used?

You can find out more about how we use your information:

- Visiting www.hra.nhs.uk/information-about-patients/
- Viewing our leaflet available from www.hra.nhs.uk/patientdataandresearch
- By asking one of the research team (see Study Coordinator details)
- By sending an email to the ELFT Data Protection Officer, Chris Kitchener at elft.dpo@nhs.net
- By ringing the DPO Office on 020 7655 4000

What will happen to the findings of this study?

We will publish the findings of this research in reports and scientific journals. When we report what you have told us we will not use your name or any other personal identifying information about you. Instead, we will replace your name with a code (number). In this way, the information you give us will be anonymised.

We will also hold some events in Newham to share our findings with the wider community. We will invite you to attend and participate in these events.

Who is organising and funding this study?

The research is being organised by Dr Michelle Heys (Chief Investigator) who is a Consultant Community Paediatrician at the East London NHS Foundation Trust. The study is funded by the Patrick Paul Foundation.

How have patients and the public been involved in this study?

We asked a parent of a child who has undergone autism assessment to give their views on all the research documents (e.g. questionnaires and information sheets). We also asked for their advice and guidance on recruitment procedures. We have also held focus groups with parents of children who have undergone autism assessment with CHAND when designing the online assessment.

Who has reviewed this study?

This study has been independently peer-reviewed and has received regulatory approvals. The study has received HRA approval from the Health Research Authority (HRA) and Favourable Ethical Opinion from the Research Ethics Committee (REC)

Further information and contact details

If you would like to talk to someone about the study please contact:

Chief Investigator:

Dr Michelle Heys,

Email: michelle.heys@nhs.net

Consultant Paediatrician and Paediatric Lead for Autism Pathway:

Dr Shermina Sayani

Email: shermina.sayani@nhs.net

Participant Information Sheet

Clinicians

Pilot clinical validation of online assessment procedure

We are inviting you to take part in a research study taking place at the Specialist Children's and Young People's Service (SCYPS) in the East London Foundation Trust. Before you decide whether to take part, it is important that you know why we are doing the study and what is involved. Please read the following information carefully.

This research study is part of a project called CHATA, which stands for Children's Autism Technology-Assisted Assessments (CHATA). The CHATA project is funded by The Patrick Paul Foundation, the Sponsor Organisation (Sponsors number: H-2019) and the Data Controller is the East London Foundation Trust.

What we're doing is trying to design a way to run autism assessments online or partially online. We are using several existing autism screening and assessment measures alongside some novel tools and trialling them as part of autism assessments for children under 5 years old.

What is the purpose of the study?

The purpose of the study is to work with parents/carers and health professionals to evaluate whether online methods of screening and assessment for autism are feasible and acceptable to the ethnically and linguistically diverse community in Newham.

We aim to develop a validated, acceptable, efficient, and feasible method of assessing autism online that, when fully implemented, will be applicable to communities throughout the UK, irrespective of their ethnic diversity and which will be tailored to surmount language barriers.

Why have I been chosen?

We are inviting clinicians at CHAND to help administer the online autism screening and assessments.

What would taking part involve?

If you decide to take part, we will ask you to deliver online autism screening and assessment to children under 5 on the CHAND waiting list and their parents. You will be given detailed instructions around how to deliver the screening and assessment by CHAND service lead, Dr Sayani, as well as other members of the CHATA research team. We will invite you to interview after delivering the online assessment(s) to understand your experiences and views on delivering autism assessments online (20-40 minutes)

Will you record the appointment and interview?

Yes. We will ask for your consent (permission) to audio and/or video record the appointments you administer and also the interview.

How much time will it take?

The time to complete online autism assessments should be approximately 1-1.5 hours per assessment and then you will be asked to take part in one 20-40 minute interview.

How many people are involved in this research?

We hope to involve around 35 parents/carers and 1-5 clinicians.

What happens if we cannot do face-to-face research due to social distancing or lockdown rules?

If we are unable to do face-to-face research, we will invite you to participate in a remote discussion or interview via phone or video call (e.g. Skype, Zoom, Microsoft Teams).

If you choose to have a video call, we will ask if you have access to a tablet, laptop, smartphone or PC computer. We will also check that you have internet access and that you can secure a private space at home. We will then email you an invitation and a link to join the discussion. If you require additional data to participate in the interview, we will reimburse you for these costs.

Do I have to take part in this research?

No. It is up to you to decide whether you would like to take part.

Will you reimburse my costs?

You will deliver the online assessment as part of your clinical care, so there will be no reimbursement for this. We will reimburse your travel costs when attending the interview, if needed. If you participate in a remote interview (e.g. on the phone or skype) we will give you a voucher to cover data costs if needed (i.e. top up mobile phone voucher) instead of travel expenses.

What are the possible benefits of taking part?

You have the opportunity to aid the development of autism assessment tools and give your feedback on how we shape the online service.

We are also able to offer you a shopping voucher to thank you for your participation.

What are the possible disadvantages of taking part?

A potential disadvantage is that you could become distressed talking about aspects of autism assessment. We will do our best to minimise any distress by creating a safe and supportive environment. A trained facilitator will be able to support you within the session and follow up afterwards for debrief.

You do not have to answer any questions if you do not feel comfortable. You can also leave the discussion at any point.

What if there is a problem?

You can contact the Study Coordinator if there is a problem. If you would like to make a formal complaint, you can contact the Chief Investigator (Dr Michelle Heys) who will follow the complaints procedure.

How will we use information about you?

If you choose to enrol in this study, we will ask you for your contact details. This includes: your name, address, mobile number and email address. This will be kept confidential. Only the Study

Coordinator will have access to your contact details. They will destroy this information at the end of the study.

Any audio and video recordings made will be kept securely. Only the research team and an external transcriber will have access to the recordings. The recordings will be destroyed at the end of the study.

A typed-up record of your answers (transcript) will be kept for a longer period. This will be stored anonymously. This means that your name, and any other identifying information, will not be linked to what you have said.

This anonymised information will be shared among members of the research team. We will also use this anonymous information in research reports and publications. In the future, it may also be shared with other researchers at other institutions.

We will destroy all copies of the anonymous study data after 10 years.

Limits to Confidentiality

If you tell us anything in the interview or discussion that makes the Study Coordinator worry that either you or your family are at risk of harm, the Study Coordinator may have to inform the Chief Investigator (Dr Michelle Heys). If this happens, the Study Coordinator will discuss this with you, before they do this.

What are your choices about how your information is used?

You can stop being part of the study at any time by letting the Study Coordinator know. You do not have to give a reason.

If you decide to withdraw, the Study Coordinator will ask what you would like us to do with your data. You can request to have your personal data (e.g. any records of your name and contact details) erased at any time. However, we will keep the research data that you have already given us (e.g. your responses in interviews and group discussions). This information will not be linked to your name or contact details. Your data will have a code number instead.

Where can you find out more about how your information is used?

You can find out more about how we use your information:

- Visiting www.hra.nhs.uk/information-about-patients/
- Viewing our leaflet available from www.hra.nhs.uk/patientdataandresearch
- By asking one of the research team (see Study Coordinator details)
- By sending an email to the ELFT Data Protection Officer, Chris Kitchener at elft.dpo@nhs.net
- By ringing the DPO Office on 020 7655 4000

What will happen to the findings of this study?

We will publish the findings of this research in reports and scientific journals. When we report what you have told us we will not use your name or any other personal identifying information about you. Instead, we will replace your name with a code (number). In this way, the information you give us will be anonymised.

We will also hold some events in Newham to share our findings with the wider community. We will invite you to attend and participate in these events.

Who is organising and funding this study?

The research is being organised by Dr Michelle Heys (Chief Investigator) who is a Consultant Community Paediatrician at the East London NHS Foundation Trust. The study is funded by the Patrick Paul Foundation.

How have patients and the public been involved in this study?

We asked a parent of a child who has undergone autism assessment to give their views on all the research documents (e.g. questionnaires and information sheets). We also asked for their advice and guidance on recruitment procedures. We have also held focus groups with parents of children who have undergone autism assessment with CHAND when designing the online assessment.

Who has reviewed this study?

This study has been independently peer-reviewed and has received regulatory approvals. The study has received HRA approval from the Health Research Authority (HRA) and Favourable Ethical Opinion from the Research Ethics Committee (REC)

Further information and contact details

If you would like to talk to someone about the study please contact:

Chief Investigator:

Dr Michelle Heys,

Email: michelle.heys@nhs.net

Consultant Paediatrician and Paediatric Lead for Autism Pathway:

Dr Shermina Sayani

Email: shermina.sayani@nhs.net
